# PP2A Phosphatase as an Emerging Viral Host Factor

**DOI:** 10.3389/fcimb.2021.725615

**Published:** 2021-08-04

**Authors:** Michal Slawomir Barski, Jordan James Minnell, Goedele Noella Maertens

**Affiliations:** Department of Infectious Disease, Section of Molecular Virology, St Mary’s Hospital, Imperial College London, London, United Kingdom

**Keywords:** PP2A (protein phosphatase 2A), B56-PP2A, LxxIxE SLiM, integrase, viral PP2A targeting

## Abstract

Protein phosphatase 2A (PP2A) is one of the most ubiquitous cellular proteins and is responsible for the vast majority of Ser/Thr phosphatase activity in eukaryotes. PP2A is a heterotrimer, and its assembly, intracellular localization, enzymatic activity, and substrate specificity are subject to dynamic regulation. Each of its subunits can be targeted by viral proteins to hijack and modulate its activity and downstream signaling to the advantage of the virus. Binding to PP2A is known to be essential to the life cycle of many viruses and seems to play a particularly crucial role for oncogenic viruses, which utilize PP2A to transform infected cells through controlling the cell cycle and apoptosis. Here we summarise the latest developments in the field of PP2A viral targeting; in particular recent discoveries of PP2A hijacking through molecular mimicry of a B56-specific motif by several different viruses. We also discuss the potential as well as shortcomings for therapeutic intervention in the face of our current understanding of viral PP2A targeting.

## Introduction

Reversible protein phosphorylation is a crucial and ubiquitous post-translational modification (PTM). One third of all proteins are thought to experience phosphorylation on the hydroxylated sidechains of serine, threonine or tyrosine residues in an equilibrium maintained by the antagonistic actions of protein kinases and phosphatases. These enzymes are classified based on their substrates as modifying either Ser/Thr or Tyr residues. Although the substrate pool is identical, the number of Ser/Thr phosphatases encoded in the human genome is vastly outnumbered by the kinases (Chen et al., 2017) and is divided into metal-dependent phosphatases (PPM), or phosphoprotein phosphatases (PPP) by virtue of sequence and fold (Chen et al., 2017). There are seven PPP families, the largest of which is the protein phosphatase 2A (PP2A), accounting for up to 1% of the total cellular protein content (Ruediger et al., 1991). PP2A has been coined the “master regulator of the cell cycle” as it regulates every stage of the cell cycle (Wlodarchak and Xing, 2016). It is known to dephosphorylate over 300 cellular substrates (Wlodarchak and Xing, 2016), and is involved in diverse cellular processes including development, cell proliferation and death, cell mobility and cytoskeletal dynamics, the cell cycle and signaling. In addition to its role in neurodevelopmental disorders (Houge et al., 2015; Loveday et al., 2015; Shang et al., 2016; Reynhout et al., 2019; Biswas et al., 2020; Lenaerts et al., 2021), PP2A is mostly known as a tumour suppressor and its inhibition caused by mutation, biochemical downregulation or drug binding is implicated in many cancers (Eichhorn et al., 2009; Ruediger et al., 2011; Ruvolo, 2016; O’Connor et al., 2020).

It is no surprise that viruses have evolved to target PP2A *via* several different mechanisms. The aim of this Mini Review is to summarise the different strategies viruses use to hijack PP2A to modulate its phosphatase activity and downstream signaling functions to subsequently enhance viral replication. This is the first review synthesizing our current understanding and the emerging role of LxxIxE motif mimicry-mediated PP2A targeting by viruses.

## Structure, Function and Regulation of PP2A

Despite their crucial role in cellular signaling, phosphatases have been, until recently, largely understudied compared to kinases. This is likely due to their complex structure and a multitude of oligomeric forms present in the cell (Shi, 2009). The PP2A phosphatase is a heterotrimer, composed of a horseshoe-like, alpha-helical HEAT repeat scaffold (A) subunit bound to the regulatory (B) and the catalytic (C) subunits. Both A and C subunits have two (α and β) variants, while the B subunit comprises four families (B55/PR55, B56/PR61, PR48/PR72/PR130 and PR93/striatin). Additionally, each family contains 2-5 isoforms and each one can have alternative splice and translational variants. PP2A exists mainly in two forms, either as the core dimer comprised of the A and C subunits, or as the heterotrimer where the core is bound to any one of the B-type regulatory subunits. It is the large number of possible unique structural PP2A permutations which exhibit diversity in both substrate specificity and subcellular localization (Janssens and Goris, 2001). Although the various B subunits recognize a similar region of A (Lenaerts et al., 2021), they lack structural or functional redundancy and are critical regulators for cell survival (Strack et al., 2004; Seshacharyulu et al., 2013).

PP2A regulates the cell cycle by affecting several critical pathways (Wlodarchak and Xing, 2016) including Wnt, mammalian target of rapamycin (mTOR), mitogen activated protein kinase (MAPK) and the phosphoinositide 3-kinase (PI3K). PP2A-B56 is also involved in the regulation of spindle assembly (Espert et al., 2014; Hayward et al., 2019), the cell cycle (Janssens and Goris, 2001), apoptosis (Janssens and Rebollo, 2012), DNA damage response (Peng and Maller, 2010) and chromosome segregation during meiosis (Bel Borja et al., 2020).

The vast range of cellular processes regulated by the different PP2A holoenzymes combined with the modular nature of holoenzyme assembly and lack of redundancy in function presents a perfect node for the deregulation of key cellular processes by tumors and viruses. Indeed, the PP2A genes and their regulators are tumor suppressors that are perturbed at a low (but significant) frequency in human cancers (Sangodkar et al., 2016). PP2A mutations are implicated in lung, breast, colorectal, among other cancers (Wang et al., 1998; Takagi et al., 2000; Ruediger et al., 2001b; Tamaki et al., 2004), where downregulation of PP2A results in transformation - yet minimal activity is essential for cell survival (Ruvolo, 2016). Concurrent with its role as a tumour suppressor, pharmacological inhibitors of PP2A catalytic function such as okadaic acid and microcystin act as potent tumor promoters (MacKintosh and MacKintosh, 1994). Such small molecule inhibitors were initially discovered from screens of natural products and are usually toxins produced by microbes and animals (McCluskey et al., 2002). For example, microcystin and nodularins were purified from blue-green algae, and okadaic acid was firstly identified in the marine sponge *Halicondria okadai* (Holmes et al., 1990; Dounay and Forsyth, 2002), while tautomycin is produced by *Streptomyces verticillatus* (MacKintosh and Klumpp, 1990). As such, these could be considered the first exogenous factors identified to affect PP2A activity.

Recent advancements in the structural and biochemical research on PP2A have allowed a much greater understanding of the mechanisms of its exogenous and endogenous regulation as well as effects of carcinogenic mutations (Xu et al., 2006; Cho and Xu, 2007; Xu et al., 2008; Wlodarchak et al., 2013; Jeong et al., 2021). Most PP2A mutations identified in cancer patients are located within PP2A Aα and Aβ subunits and affect the binding of other PP2A subunits to the scaffold (Ruediger et al., 2001a; Sablina et al., 2007). Post-translational modifications of the catalytic and scaffold subunits such as phosphorylation and methylation modulate the activity of PP2A in a similar way, also affecting the association with regulatory subunits (Janssens and Goris, 2001; Janssens and Rebollo, 2012; Seshacharyulu et al., 2013). Similarly, phosphorylation of B subunits regulates the sub-cellular localization of the holoenzyme affecting its substrate repertoire (Strack et al., 1998).

## PP2A is Ubiquitously Targeted and Modulated by Viruses

**Figure 1:**
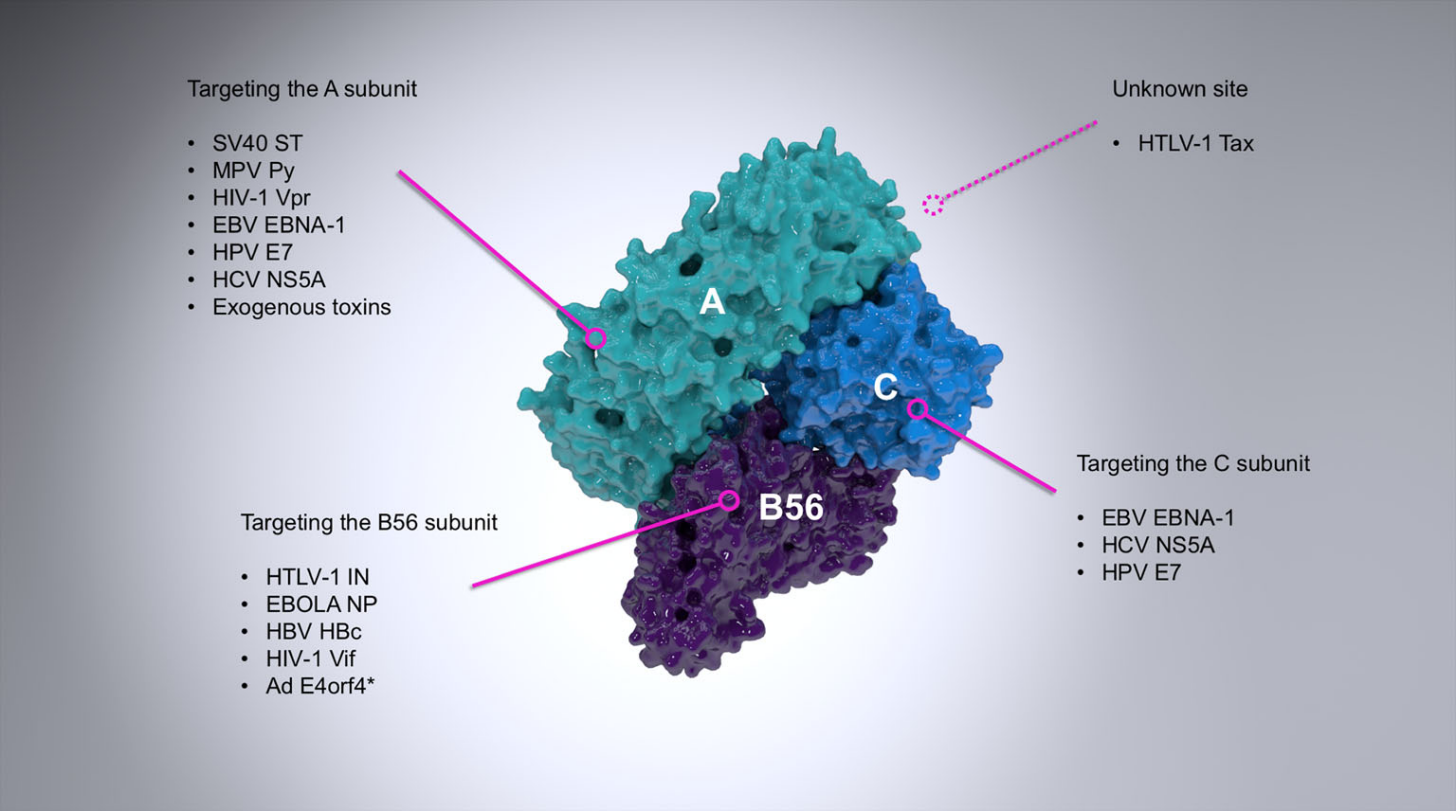
A summary of the most important examples of PP2A targeting by viruses. PP2A is a heterotrimer composed of the scaffold (A; cyan), regulatory (B56; purple) and catalytic (C; light blue) subunits. The figure shows a B56-containing PP2A holoenzyme (PDB ID: 2IAE). Viral proteins interact with PP2A *via* different subunits, as indicated where it is known. The vast majority of viral proteins known to interact with the regulatory subunit of PP2A target exclusively B56, although the adenovirus E4orf4 (asterisked above) can also target B55 subunits (Kleinberger, 2020) and some other viral proteins are known to associate exclusively with other regulatory subunits (for example the polymerase of rinderpest virus (Sleeman and Baron, 2005).

Numerous proteins from a wide range of viral families have already been identified to interact with and modulate the activity of PP2A (**Figure 1**, and extensively reviewed by (Guergnon et al., 2011)). The first known case was the small T antigen (sT) of the oncogenic simian virus 40 (SV40). sT and large T antigen (LT) of SV40, merkell cell polyomavirus (MCV), or Murine polyoma virus (Py) are alternatively-spliced oncoproteins which play a role in the transformation of infected cells (Rundell and Parakati, 2001; Yu et al., 2001; Kwun et al., 2009). This property of sT, however, is fully reliant on its binding to the scaffold subunit of PP2A (Yang et al., 1991; Ruediger et al., 1992), which displaces the regulatory subunit (Pallas et al., 1990; Chen et al., 2004) and therefore inhibits PP2A-mediated dephosphorylation of many substrates (with the known exception of histone H1) and promotes viral (DNA) replication (Scheidtmann et al., 1991; Sablina et al., 2007; Kwun et al., 2009; Bollag et al., 2010; Bhat et al., 2020). A close dissection of this interaction has been made possible through the crystal structure of the SV40 sT in complex with the Aα subunit of PP2A (Cho et al., 2007). Conversely, the West Nile virus capsid (WNVC) protein upregulates PP2A-mediated activity hereby inhibiting AP-1 dependent transcription (Hunt et al., 2007).

Several other oncogenic viruses target PP2A in order to inhibit its downstream pro-apoptotic signaling, therefore leading to transformation and immortalization. Examples include the E7 protein of human papillomaviruses (HPV) (Pim et al., 2005) or the leader protein (EBNA-LP) encoded by the Epstein-Barr virus (EBV) (Garibal et al., 2007). At the same time, other viruses target PP2A to drive apoptosis, such as the E4orf4 protein of adenovirus (Kleinberger and Shenk, 1993) and the Py sT-antigen (Bhat et al., 2020).

The innate immune response is the first line of defense upon pathogen invasion. PP2A inhibits this process by dephosphorylating interferon regulatory transcription factor 3 thus blocking expression of interferon stimulated genes (Wang et al., 2020a). Many viruses depend on (Xu et al., 2019), or exploit this process, either by stimulating (Zan et al., 2020), or upregulating PP2A (Duong et al., 2004; Christen et al., 2007) thereby blocking type I IFN response in favor of increasing viral replication.

The two retroviruses of clinical importance: human immunodeficiency virus type 1 (HIV-1) and human T-lymphotropic virus type 1 (HTLV-1) have also evolved several independent mechanisms of PP2A hijacking. The Vpr protein of HIV-1 associates with the scaffold subunit of PP2A (Godet et al., 2010) while HIV-1 Vif targets the PP2A regulatory B56 subunit for degradation (Greenwood et al., 2016; Naamati et al., 2019; Marelli et al., 2020; Nagata et al., 2020). Both processes act independently to lead to G2/M cell cycle arrest. The transactivator protein (Tax) of HTLV-1 is one of the most potent oncogenes known (Mohanty and Harhaj, 2020) and associates with an unidentified region of PP2A as part of a ternary complex with IKKγ (Fu et al., 2003; Merling et al., 2007). Inhibition of PP2A activity prevents dephosphorylation of IKKγ which leads to activation of IKKγ-regulated genes and ultimately cell cycle arrest. HTLV-1 integrase (IN) associates with the B56 subunit to enhance infection and might play a role in integration site targeting (Maertens, 2016; Barski et al., 2020; Bhatt et al., 2020). The relevance of cell senescence induced by both HIV-1 and HTLV-1 in the context of infection remains unknown.

It remains to be understood how viral hijacking of PP2A can lead to such vastly different biochemical and phenotypic events. Unfortunately, the structure-driven mechanistic understanding of these processes is still scarce, with the aforementioned sT:Aα and the IN:B56γ (described below) – the only viral protein complexes resolved with PP2A thus far.

## Emerging Role of Viral PP2A Targeting *via* LxxIxE Motif Mimicry

An emerging mechanism of PP2A binding employed by viruses relies on mimicking a short linear motif (SLiM) used by endogenous substrates and partners of PP2A to bind to all isoforms of the B56 regulatory subunit (Hertz et al., 2016; Wang et al., 2016). The SLiM of the consensus sequence LxxIxE (where ‘x’ can be any amino acid) is located on intrinsically disordered regions of many PP2A endogenous partners and becomes fixed upon binding to a highly-conserved groove between the third and fourth HEAT repeat in the centre of the concave face of the B56 subunit of PP2A. Structural studies focusing on BubR1 and RepoMan have identified the interaction interface largely defined by the Leu pocket (B56 residues K183, T184, H187, R188, E226, I227) and Ile pocket (B56 residues H187, Y190, I227, I231) (Wang et al., 2016). Interactions, both hydrophobic and electrostatic in nature, involve every residue of the LxxIxE SLiM and SLiM point mutations weaken the interaction. At the same time, Wang et al. showed that the presence of a phosphorylated serine following the SLiM leucine significantly enhances binding through a salt bridge interaction with R188. The limitation of the aforementioned studies is the use of short peptides mimicking the SLiM region rather than entire domains/subunits.

We recently discovered through proteomic mass spectrometry approaches that PP2A-B56 is a functional binding partner of the deltaretroviral integrase enzyme (Maertens, 2016). IN catalyses the insertion of reverse-transcribed viral DNA (vDNA) into host chromatin which is a key step in the deltaretroviral replication cycle. *In vitro* strand-transfer activity of deltaretroviral IN was largely stimulated in the presence of B56. Mutational scanning identified the B56 region encompassing HEAT repeats 3 and 4 critical to IN binding and strand-transfer stimulation. The long linker between the IN catalytic domain (CCD) and the C-terminal domain (CTD) encompasses the LxxIxE sequence – a *bona fide* B56-targeting motif. Indeed, every single point mutation within the simian T-cell lymphotropic virus type 1 (STLV-1) IN SLiM (L213A, P214A, P215A/P217A, I216A, E218A) reduced IN binding to B56 and *in vitro* strand-transfer activity stimulation in presence of B56 (Barski et al., 2020). Conversely, B56 mutations L194A, R197A located in the LxxIxE-binding groove also prevented this functional interaction (Maertens, 2016).

**Figure 2:**
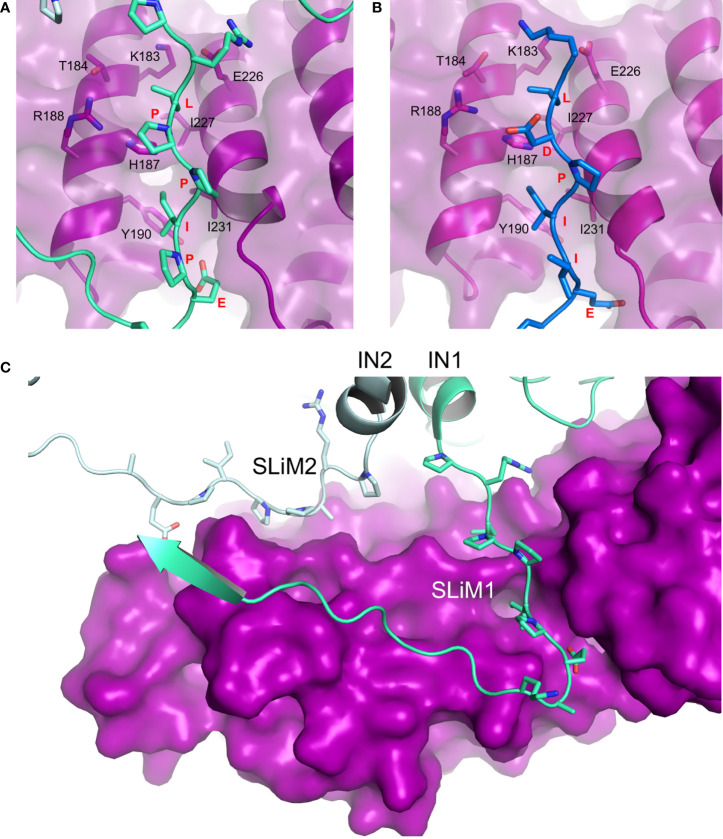
Hijacking of PP2A *via the* B56-binding LxxIxE SLiM. **(A)** Details of LPPIPE IN SLiM (equivalent to SLiM1 in panel C; green) binding to the canonical LxxIxE-binding groove on B56 (purple). Interacting residues are shown as sticks (PDB ID: 6Z2Y). **(B)** BUBR1 LDPIIE SLiM (also equivalent to SLiM1 in panel C; blue) binding to the same site on B56 (purple) (PDB ID: 5JJA). In this case, the aspartate was used as a phosphomimetic. The conformational overlap between residues of the SLiMs and the binding groove in both structures is evident. **(C)** Deltaretroviral integrase (IN) dimer contributes two identical SLiM regions to bind one molecule of B56 (purple) at two different sites. Each IN chain (IN1 and IN2) is coloured with a different shade of green (PDB ID: 6Z2Y, EMDB: 11052). PyMOL software was used for analysis.

The X-ray crystallographic structure of deltaretroviral human lymphotropic virus type-1 (HTLV-1) IN residues 200-297, encompassing the LxxIxE SLiM, and B56 revealed a binding interface akin to that of BubR1 and RepoMan (**Figures 2A, B**), yet devoid of the phosphoserine-R188 interaction (Barski et al., 2020). Our understanding of the IN-PP2A binding was then largely expanded by two independent studies by Barski et al. (2020) and Bhatt et al. (2020) who have respectively reported structures of STLV-1 and HTLV-1 intasomes (IN:vDNA supramolecular intermediates) in complexes with B56. Both structures corroborated observations from the co-crystal complex but also identified a previously-unknown ligand binding site on B56. The LxxIxE SLiM seems to have evolved strategically to exploit the oligomeric structure of the intasome in order to further stabilize the IN:B56 complex by repurposing the LxxIxE SLiM on a neighboring IN subunit (**Figure 2C**). Effectively, the two SLiMs – contributed by a dimer of IN – bind two separate sites on B56. The secondary site runs perpendicular to the primary SLiM binding groove and involves residues E78, T81, H82, N83 and R143 located on the solvent-exposed surface of HEAT repeats five and six.

Although the exact role of PP2A in deltaretroviral integration has not been discerned yet, it is an essential host factor for viral replication - shRNA knockdown of B56γ significantly reduced HTLV-1 infectivity (Bhatt et al., 2020). Even though PP2A does not directly associate with chromatin, it dephosphorylates many transcription factors and chromatin modifiers (Eichhorn et al., 2009; Shanker et al., 2013) including those whose binding sites were found in the vicinity of HTLV-1 integration sites (Melamed et al., 2013). Binding of IN to PP2A does not occlude the active site (Barski et al., 2020; Bhatt et al., 2020) suggesting IN could modulate PP2A catalytic activity to dephosphorylate itself or other targets, although clearly the presence of phosphorylated IN is not essential for binding. PP2A might also play a role in targeting the pre-integration complex to the site of integration, as has been known for IN-associated host factors in other retroviral families (Kvaratskhelia et al., 2014).

Interestingly, Wang et al. recently also identified a binding site on B56 for yet another motif. This “acidic patch” on B56 is located in close proximity to the primary LxxIxE SLiM binding sites utilized by deltaretroviral INs (Wang et al., 2020b). In the structures of STLV/HTLV intasome:B56 complexes the “acidic patch” identified by Wang et al. is not occupied. It is argued that a subset of LxxIxE-containing endogenous partners and substrates of PP2A need an additional site as the SLiM binding to the primary site on B56 only confers micromolar-range affinities. It is tempting to speculate that the divergence in mechanisms of binding between endogenous and deltaretroviral PP2A binders (that have been demonstrated so far) could be exploited for antiviral drug design to avoid side effects caused by inhibition of PP2A dephosphorylation activity in key cellular pathways. Such an approach may also mean that the same inhibitor could be used against more than one virus. Additionally, targeting a site on a host protein, rather than a viral protein minimizes the problem of drug resistance.

Although mostly known for its ability to target APOBEC3 restriction factors for proteasomal degradation (Desimmie et al., 2014; Harris and Dudley, 2015), intense studies on the HIV-1 Vif accessory protein revealed that the cell cycle arrest induced by Vif occurs through its targeted degradation of B56 (Greenwood et al., 2016; Marelli et al., 2020; Nagata et al., 2020; Salamango and Harris, 2020). Interestingly, although Vif does not encode an LxxIxE SLiM motif, co-expression of an LxxIxE-like peptide inhibitor known to outcompete B56 binders reduced degradation of B56 in a dose-dependent manner (Salamango et al., 2020) suggesting that Vif recognition by B56 involves residues surrounding the LxxIxE SLiM binding pocket.

The panel of LxxIxE SLiM-utilising viruses includes filoviruses (Hertz et al., 2016) and it has been shown that the most clinically-important filovirus, the Ebola virus (EBOV), associates with B56 *via* the SLiM located on its nucleoprotein (NP). Although structural details of this interaction are still missing, Kruse et al. determined that NP associates with PP2A-B56 to dephosphorylate VP30, an EBOV-encoded protein and binding partner of NP (Kruse et al., 2018). VP30 is an EBOV-specific transcription factor, regulated by phosphorylation (Takamatsu et al., 2020), and essential for initiation of viral transcription (Tigabu et al., 2018). The NP LxxIxE, and hence its interaction with B56, was found to be required for EBOV transcription, but not replication. Interestingly, phosphorylation of VP30 increases its affinity for NP (Biedenkopf et al., 2013) which in turn increases its chances of PP2A-mediated dephosphorylation – likely forming a negative feedback loop to balance EBOV transcription and replication.

Another example of viral B56 recruitment *via* the LxxIxE SLiM was recently discovered in the hepatitis B virus (HBV) capsid or core protein (HBc) (Xi et al., 2021). HBc is a multifunctional protein taking part in every step of HBV replication: from its role as the nucleocapsid to reverse transcription, to vDNA nuclear import and packaging (Nassal, 1992; Wynne et al., 1999; Liu et al., 2015). HBc is regulated through phosphorylation of its CTD – supporting different steps of the HBV viral life cycle depending on the level of CTD phosphorylation (Ludgate et al., 2016; Luo et al., 2020). The SLiM is located on the NTD-CTD linker whose truncation was previously reported to affect multiple aspects of HBV replication as well as NTD assembly and the phosphorylation of the CTD (Liu et al., 2018). Point mutations within the HBc LxxIxE caused multiple pleiotropic effects in the HBV life cycle resulting in reduced HBV infectivity. Surprisingly, the HBc SLiM slightly diverges from the canonical LxxIxE consensus sequence, encoding a leucine instead of the isoleucine (LSTLPE). Although no direct interaction between HBc and B56 was identified, it remains possible that the interaction is very transient in nature and/or relies on varying degrees of HBc phosphorylation.

## Conclusions

PP2A is a promising anti-cancer drug target (O’Connor et al., 2018; Leonard et al., 2020) and PP2A targeted agents are currently used in combination therapy in cancer (Mazhar et al., 2019). Here, we have synthesized efforts of the last few years in broadening our understanding of viral PP2A targeting; particularly *via* the LxxIxE-mediated B56 binding. Utilizing PP2A’s vast repertoire of functions and partners seems to be crucial for many clinically-relevant viruses such as Ebola, West Nile virus, HIV-1, HTLV-1 and many oncogenic viruses. It is imperative to further our understanding of PP2A hijacking and once more structural and mechanistic details of this phenomenon are obtained, PP2A might present an opportunity for antiviral pharmacological interventions.

## Author Contributions

MB, JM and GM wrote the Review. All authors contributed to the article and approved the submitted version.

## Funding

MB, JM and GM are funded by the Wellcome Trust ([Investigator Award 107005 to GM]).

## Conflict of Interest

The authors declare that the research was conducted in the absence of any commercial or financial relationships that could be construed as a potential conflict of interest.

## Publisher’s Note

All claims expressed in this article are solely those of the authors and do not necessarily represent those of their affiliated organizations, or those of the publisher, the editors and the reviewers. Any product that may be evaluated in this article, or claim that may be made by its manufacturer, is not guaranteed or endorsed by the publisher.
